# Occurrence of extended- spectrum β-lactamase harboring *K. pneumoniae* in various sources: a one health perspective

**DOI:** 10.3389/fcimb.2023.1103319

**Published:** 2023-05-23

**Authors:** Nafeesa Yasmeen, Bilal Aslam, Liang-xing Fang, Zulqarnain Baloch, Yahong Liu

**Affiliations:** ^1^College of Veterinary Medicine, South China Agricultural University, Guangzhou, China; ^2^Institute of Microbiology, Government College University, Faisalabad, Pakistan; ^3^Faculty of Life Science and Technology, Kunming University of Science and Technology, Kunming, Yunnan, China

**Keywords:** *K. pneumonia*, extended spectrum beta-lactamases, prevalence, one health, Pakistan

## Abstract

This study was designed to investigate the occurrence and dissemination of extended-spectrum β-lactamase (ESBL) harboring *Klebsiella pneumoniae* in various ecological niches under the one health approach. A total of 793 samples were collected from animals, humans, and the environment. The findings of the study revealed the occurrence of *K. pneumoniae* as follows: animals (11.6%), humans (8.4%), and associated environments (7.0%), respectively. A high occurrence rate of ESBL genes was found in animals compared to human and environmental isolates. A total of 18 distinct sequence types (STs) and 12 clonal complexes of *K. pneumoniae* were observed. Overall, six STs of *K. pneumoniae* were identified in commercial chickens, and three were found in rural poultry. The majority of *K. pneumoniae* STs found in this study were positive for *bla*SHV, while the positivity of other ESBL-encoding genes combinations was different in different STs. The high occurrence rate of ESBL-harboring *K. pneumoniae* found in animals as compared to other sources is alarming and has the potential to be disseminated to the associated environment and community.

## Introduction

At present, it is well recognized that antimicrobial resistance (AMR) happens in three interconnected sectors: human, animal, and environmental segments (Aslam et al., 2021). Therefore, AMR is described by the “One Health” case study, which demonstrates the essential coordinated role of animal, human, and environmental experts in reducing the worldwide spread of this health crisis. However, the specific role of these sectors for development, transmission, and antibiotic-resistant organism perseverance is still vague and requires more attention to find out a sustainable solution to this global health concern.

The emergence of multidrug- resistant (MDR) bacteria, particularly *Enterobacteriaceae*, is a major cause of increased mortality and morbidity in humans and livestock (de Been et al., 2014). The first report of plasmid-encoded β-lactamases capable of hydrolyzing the extended-spectrum cephalosporins was published in 1983 (Knothe et al., 1983). In Gram-negative bacteria, broad-spectrum enzymes such as TEM-1 and SHV-1 arose following the introduction of first- and second-generation cephalosporins. Subsequently, expanded-spectrum β-lactam antibiotics were introduced that were refractory to hydrolysis by these enzymes. They confer resistance to most β-lactam antibiotics, including expanded-spectrum cephalosporins and monobactams, but not to carbapenems and cephamycins (Castanheira et al., 2021). ESBL-producing Gram-negative pathogens are now usual in both hospital and community settings (Pitout et al., 2005).

The epidemiology of ESBLs is complex and rapidly changing. The incidence of ESBLs producing *Klebsiella pneumoniae* is increasing worldwide, but its detection rate varies. Until 2000, ESBL types were predominantly TEM and SHV types, which were most commonly detected in *K. pneumoniae* (Ribeiro da Cunha et al., 2019). However, in the 2000s, CTX-M-type ESBLs constituted the majority of ESBLs in the community. In Latin America, from 2008 to 2010, the detection rates of ESBL-producing *Klebsiella* spp. were 60.4%, 59.2%, 49.9%, and 33.3% in Argentina, Chile, Brazil, and Mexico, respectively (Oka et al., 2022).

Pakistan is the world’s fifth largest populated country. There has been a consistent increase in ESBL-producing pathogens in Pakistan (Chen et al., 2011). In Pakistan, most research studies are conducted on clinical samples in well-developed areas such as Punjab and Sindh (Kumarasamy et al., 2010; Chen et al., 2011; Aslam et al., 2018; Baloch et al., 2019; Mohsin et al., 2019; Chaudhry et al., 2020). Very limited studies have been reported related to food animals, particularly poultry (Aslam et al., 2018; Baloch et al., 2019; Mohsin et al., 2019; Chaudhry et al., 2020). Here, we report the occurrence of extended-spectrum β-lactamase (ESBL) harboring *K. pneumoniae* in various sample sources under the One Health approach, which would be beneficial to apprehend the current health scenario in local health care settings.

## Materials and methods

### Sample collection

In this study, a total of 793 samples were collected from January 2019 to October 2019 from 430 animals, 320 humans, and 43 from their associated environments in Punjab, Pakistan. Convenient sampling was done throughout the study to collect samples. The samples, i.e., fecal samples (human and animals), farm wastewater, feed, etc., were collected from farms, villages, and associated environments and stored in cool boxes containing/having ice packs with 4°C temperature and sent to the laboratory for further processing.

### Isolation and identification of *K. pneumoniae*

In brief, fecal swabs and feed samples were dispensed into sterilized glass tubes containing 1 ml of PBS followed by streaking on nutrient agar plates. Other samples were streaked directly on the nutrient agar plates. Plates were incubated for 24 h at 37°C. Afterward, selective media named HiChrom *Klebsiella* Selective agar (Himedia^®^) and MacConkey’s agar were used for the isolation of *K. pneumoniae*. Biochemical characterization of the isolates was done by the API 20E Kit and VITEK identification system (bioMérieux).

### Antimicrobial susceptibility testing

AST was performed under the guidelines of Clinical and Laboratory Standards Institute (CLSI) guidelines (CLSI, 2013), veterinary CLSI (VET01-A4/VET01-S2), Clinical and Laboratory Standards Institute (CLSI, 2015: M100-S25) (Institute CaLS, 2013). The studied antibiotics include amikacin (AK), cephalexin (CN), tobramycin (TOB), ampicillin (AMP), amoxicillin/clavulanate (AMC), piperacillin (PIP), tazobactum (TZP), cefoperazone + sulbactam (SCF), cefepime (FEP), cefotaxime (CTX), ceftriaxone (CRO), imipenem (IMP), meropenem (MEM), ciprofloxacin (CIP), levofloxacin (LEV), trimethoprim-sulfamethoxazole (SXT), minocycline (MIN), ceftolozane/tazobactum (CT), polymyxin (PB), and tigecycline (TGC). *E.coli* ATCC 25922 was used as the quality- control strain.

### Detection of extended-spectrum β-lactamases

Phenotypic confirmation of EBLS production was done through DDST, as performed earlier. Concisely, cefotaxime (30 μg) disc alone and combined with clavulanic acid (30:10 μg) with 20 mm of distance were placed, respectively. Afterward, isolates were incubated, and results were interpreted, i.e., the difference of ≥5 mm in the zone of inhibition was taken as positive.

Subsequently, ESBL resistance genes were detected in the isolates. For that purpose, strains were cultured in brain heart infusion broth at 37 °C overnight. Genomic DNA was extracted from samples using a TIANamp Bacterial DNA extraction kit (DNA Kit DP302, Beijing, China) following the manufacturer’s instructions. The DNA quality was determined using A NanoDrop-2000 spectrophotometer (Thermo Fisher Scientific, Austin, TX, USA).

For the detection of ESBLs, i.e., *bla*SHV, *bla*TEM, *bla*CTX–M–1, *bla*CTX–M–2, *bla*CTX–M–8, *bla*CTX–M–9, *bla*CTX–M–14, and *bla*CTX–M–15, PCR was performed with conditions as follows: initial denaturation at 95°C for 8 min, followed by 29 cycles (denaturation at 95°C for 30 s, annealing at 55°C for 30 s, extension at 72°C for 30 s) and final extension at 72°C for 7 min. Subsequently, the 1.5% agarose gel electrophoresis was performed to visualize the PCR products and interpreted by the gel doc system (BioRad^®^, USA).

### Multilocus sequence typing (MLST)

The MLST was carried according to the Pasteur MLST (https://bigsdb.pasteur.fr/klebsiella/) scheme by the amplification of seven housekeeping genes of *K. pneumoniae* (*rpoB*, *infB*, *gapA*, *pgi*, *mdh*, *phoE*, and *tonB*). The PCR was carried out, by making up a 50 μl reaction mixture comprising of 5 μl of 2X PCR Master Mix (Thermo-Scientific™, USA), each primer (10 μM) containing 1 and 2 μl of sample DNA. The respective gene- acquired PCR product was visualized on ethidium bromide–stained 1% agarose gel. The amplicons of housekeeping genes were sequenced with respective primers. Subsequently, aligned sequences were compared against the MLST database for *K. pneumoniae* according to the pasture scheme.

## Results

### Occurrence of extended-spectrum β-lactamase harboring *K. pneumoniae* in various sources

In general, a total of 80 (10%) *K. pneumoniae* isolates were recovered from various sources. The source-wise occurrence of ESBL harboring *K. pneumoniae* estimated in the study was animal source (11%), environment source (7%), and human source (8%) (**Table 1**). Among different sample types in animal sources, the highest detection rate (16%) was observed in chicken samples whereas, among different sample types in the environment, the highest detection rate (12%) was found in farm waste. Slaughterhouse workers showed the highest detection rate (12.5%) of *K. pneumoniae* among various samples collected in the human category (**Table 1**).

**Table 1:** Occurrence of extended-spectrum β-lactamase (ESBL) harboring *K. pneumoniae* isolates among various sources.

Origin of sample	Samples	Number- resistant isolates	ESBL-resistance genes-positive *K. pneumoniae* (%)						
			*bla*_SHV_	*bla*_TEM_	*bla*_CTX-M_	*bla*_CTX-M-1_	*bla*_CTX-M-8_	*bla*_CTx-M-9_	*bla*_CTX-M-15_
Animals	430	50 (11.6)	46 (10.7)	47 (10.9)	39 (9.1)	27 (6.3)	11 (2.5)	12 (2.8)	13 (3.0)
Commercial chicken	280	44(15.7)	41 (14.6)	41 (14.6)	34 (12.1)	24 (8.6)	9 (3.2)	10 (3.6)	12(4.3)
Cow and buffalo	70	2 (2.8)	1(1.4)	2 (2.8)	1(1.4)	1(1.4)	1(1.4)	0	0
Sheep and goat	80	4 (5.0)	4(5.0)	4(5.0)	4(5.0)	2(2.5)	1(1.2)	2(2.5)	1(1.2)
Environment	43	3(7.0)	3(7.0)	3(7.0)	3(7.0)	2(4.6)	1(2.3)	1(2.3)	0
Feed	20	1(5)	1(5)	1(5)	1(5)	1(5)	0	0	0
Water	15	1(6.7)	1(6.7)	1(6.7)	1(6.7)	1(6.7)	1(6.7)	0	0
Waste	8	1(12.5)	1(12.5)	1(12.5)	1(12.5)	0	0	1(12.5)	0
Human	320	27 (8.4)	22 (6.9)	23 (7.2)	19 (5.9)	16 (5)	7(2.2)	2(0.6)	3(0.9)
Farm/Household worker	112	11(9.8)	8(7.1)	10 (8.9)	9(8.0)	7(6.2)	2(1.8)	1(0. 9)	1(0. 9)
Slaughter worker	8	1(12.5)	1(12.5)	0	1(12.5)	1(12.5)	1(12.5)	0	0
Unhealthy individuals	100	12(12.0)	11(11.0)	10(10.0)	7(7.0)	6(6.0)	3(3.0)	1(1.0)	2(2.0)
Health individual	100	3(3.0)	2(2.0)	3(3.0)	2(2.0)	2(2.0)	1(1.0)	0	0
Total	793	80(10.1)	71 (8.9)	73 (9.2)	61(7.7)	45 (5.7)	19(2.4)	15(1.9)	16(2.0)

### Antibiotic resistance profiling

Overall, all isolates (n = 80) were found resistant to the majority of studied antibiotics except tigecycline, polymyxin, and ceftolozane/tazobactam (**Supplementary Table**). A total of 79 isolates (98.7%) were resistant to cephalexin (CN), and trimethoprim-sulfamethoxazole (SXT). A total of 78 isolates (97.5%) were resistant to tobramycin (TOB). A total of 75 (93.7%) isolates were resistant to amikacin hydrate (AK). A total of 74 isolates (92.5%) were resistant to minocycline (MIN). There were 100% isolates that were resistant to all antibiotics including ampicillin (AMP) (100%), amoxicillin/clavulanate (AMC) (100%), piperacillin (PIP) (100%), piperacillin/tazobactam (TZP) (100%), cefoperazone + sulbactam (SCF (100%), cefepime (FEP) (100%), cefotaxime (CTX)(100%), ceftriaxone (CRO) (100%), imipenem (IMP) (100%), meropenem (MEM) (100%), ciprofloxacin (CIP) (100%), and levofloxacin (LEV) (100%).

### Detection of extended-spectrum β-lactamase genes

**Figure 1:**
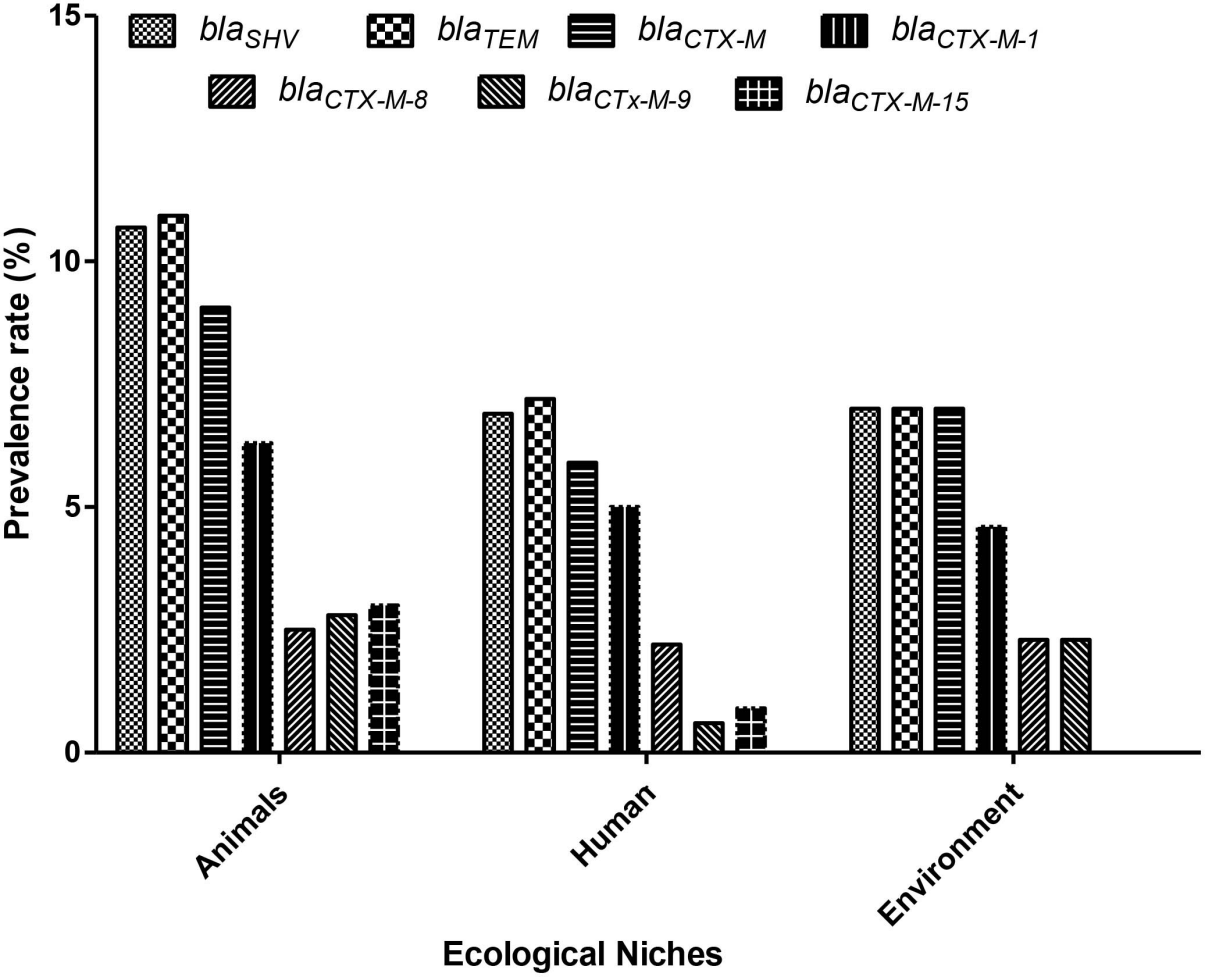
Prevalence of extended-spectrum β-lactamase (ESBL)– resistant genes among various ecological niches in Pakistan. X-axis: Ecological niches; Y-axis: ESBL-resistance gene-positive *K. pneumoniae* (%) (n = 80).

All *K. pneumoniae* strains isolated from animals, environment, and human samples were positive for various ESBL genes like *bla*SHV, *bla*TEM, *bla*CTX-M, *bla*CTX-M-1, *bla*CTX-M-8, *bla*CTX-M-9, and *bla*CTX-M-15 (**Table 1**). Among them, *bla*TEM and *bla*SHV showed the highest (94% and 92%) detection rates in animal samples compared to the other genes like *bla*CTX-M (78%), *bla*CTX-M-1 (54%), *bla*CTX-M-15 (26%), *bla*CTX-M-9 (24%), and *bla*CTX-M-8 (22%). Animal samples showed a high ESBL detection rate as compared to human and environmental isolates (**Figure 1**). In the case of environmental samples, *bla*SHV, *and bla*TEM, were present in all (100%) isolates while other ESBL genes were detected variably. Likewise, the most detected ESBL genes among human sources were *bla*TEM and *bla*SHV, 85% and 81%, respectively, whereas the least detected gene was *bla*CTX-M-9, i.e., 7% (**Table 1**).

### Co-occurrence of extended-spectrum β-lactamase genes among various samples

Overall, various combinations were observed that showed the co-occurrence of ESBL genes among these isolates. However, maximum isolates showed the co-existence of *bla*TEM and *bla*SHV along with *bla*CTX-M and its variant *bla*CTX-M-1. Isolates from animal origin showed the co-occurrence pattern with *bla*CTX-M, while isolates from human sources were observed with the occurrence of *bla*TEM and *bla*SHV with *bla*CTX-M-1. Likewise, environment samples also displayed the co-occurrence pattern of ESBL genes.

### Sequence types

**Figure 2:**
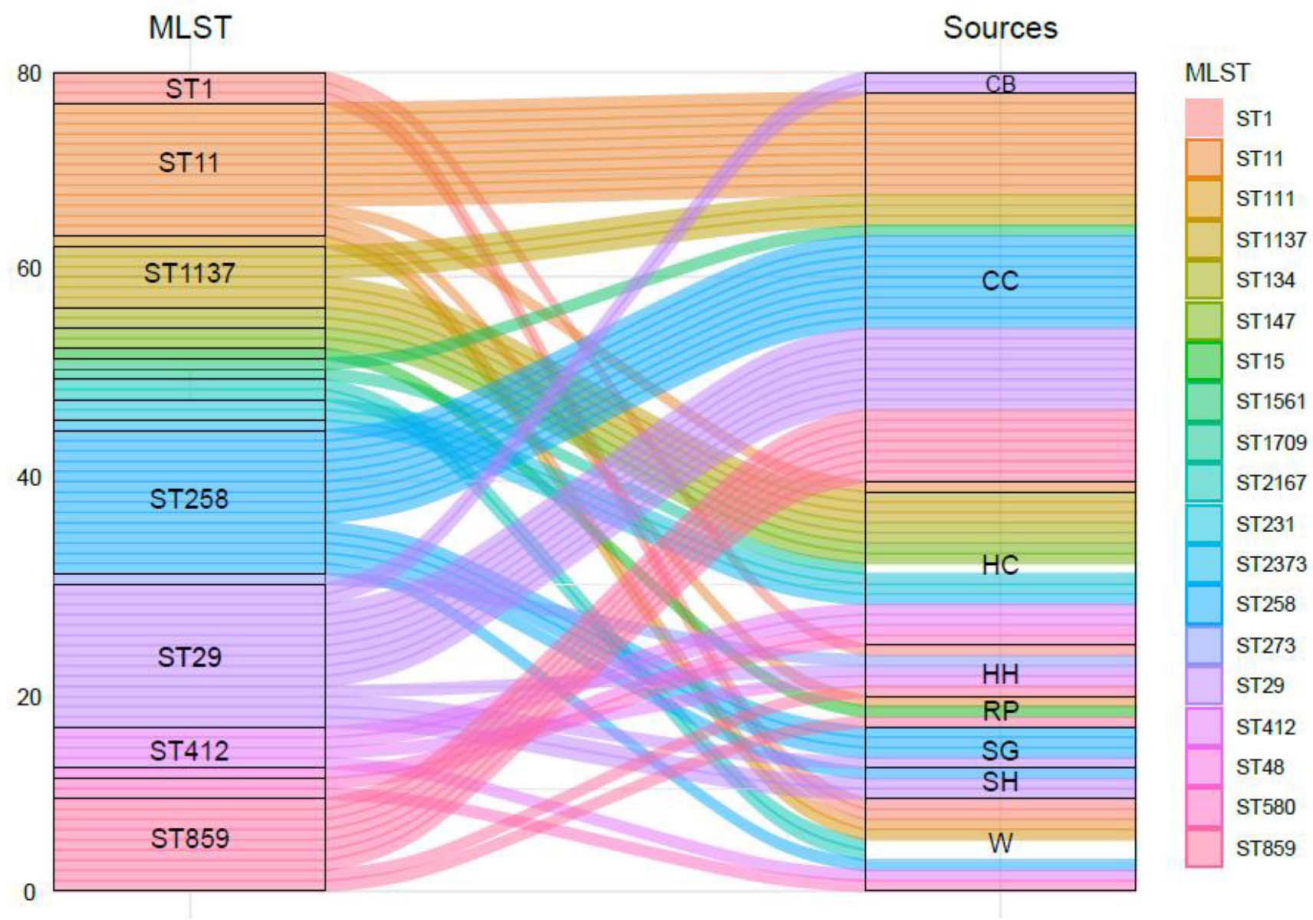
The genetic diversity of ESBL-producing *K. pneumoniae* isolates from human, animal, and associated environments. CB, cow and buffalo; CC, commercial chicken; HC, human clinic; HH, household; RP, rural poultry; SG, sheep and goat; SH, slaughter house; W, water.

Overall, a total of 18 distinct sequence types (STs) and 12 clonal complexes (CCs) of *K. pneumoniae* were detected in the study. MLST results revealed that ST29 and ST258 were found the most prevalent (17%) STs among all isolates of the study followed by ST11 (16%), whereas some of the least observed STs include ST147 and ST273 (2%). Details of STs are given in **Table 2**. A total of six distinct STs of *K. pneumoniae* were identified in commercial chickens, such as ST11 (n = 10), ST258 (n = 9), ST29 (n = 8), ST859 (n = 7), ST1137 (n = 3), and ST1561 (n = 1) (**Figure 2**), while three distinct STs of *K. pneumoniae*, ST11, ST15, and ST859, were found in rural poultry. Interestingly, ST15 was only found in rural poultry, and ST1561 was only found in commercial chickens. ST11 was the only sequence found in feed and farm waste. Various STs found in human sources were ST48, ST134, ST147, ST231, ST273, ST412, ST580, ST1137, and ST1709 (**Figure 2**). Additionally, a total of 12 CCs were found among them. CC11 and CC147 were the two most frequent CCs found in the study.

**Table 2:** Source- wise detection details of sequence types along with the co-occurrence of ESBL genes among various isolates.

Sequence type	Clonal complex	Isolates	Sources^#^	Number of antibiotics that *K. pneumoniae* were resistant (isolates)	ESBL resistance genes
**ST1**	1	3	W (2), HH (1)	17 (3)	bla_SHV_ (3), bla_TEM_ (2), bla_CTX-M_ (2) bla_CTX-M-1_ (2), bla_CTX-M-15_ (1)
**ST11**	11	13	CC (10), RP (1), Feed (1), Waste (1)	16 (1), 17 (11), 19 (1)	bla_SHV_ (12), bla_TEM_ (13), bla_CTX-M_ (10), bla_CTX-M-1_ (7), bla_CTX-M-8_ (4), bla_CTX-M-9_ (3), bla_CTX-M-15_ (5)
**ST15**	15	1	RP (1)	17 (1)	bla_SHV_, (1) bla_CTX-M-1_ (1), bla_CTX-M-15_ (1)
**ST111**	111	1	W (1)	17 (1)	bla_TEM_ (1), bla_CTX-M_ (1), bla_CTX-M-8_ (1)
**ST29**	29	14	SH (2), C&B (2), S&G (1), HH (1), CC (8)	17 (11), 16 (2), 15 (1)	bla_SHV_ (12), bla_TEM_ (13), bla_CTX-M_ (10), bla_CTX-M-1_ (7), bla_CTX-M-8_ (2), bla_CTX-M-9_ (2), bla_CTX-M-15_ (2)
**ST48**	48	1	HC (1)	17 (1)	bla_SHV_ (1), bla_TEM_ (1), bla_CTX-M_ (1), bla_CTX-M-1_ (1), bla_CTX-M-15_ (1)
**ST134**	292	2	HC (2)	17 (2)	bla_SHV_ (2), bla_TEM_ (2), bla_CTX-M_ (1), bla_CTX-M-8_ (1), bla_CTX-M-9_ (1)
**ST147**	147	2	HC (2)	16 (1), 15 (1)	bla_SHV_ (1), bla_TEM_ (1), bla_CTX-M_, (2), bla_CTX-M-1_ (2), bla_CTX-M-8_ (1)
**ST231**	231	2	HC (2)	17 (1), 16 (1)	bla_SHV_ (2), bla_TEM_ (2)
**ST258**	258	14	SH (1), S&G (3), Water (1), CC (9)	15 (1), 16 (1), 17 (12)	bla_SHV_ (14), bla_TEM_ (14), bla_CTX-M_ (13), bla_CTX-M-s1_ (9), bla_CTX-M-8_ (4), bla_CTX-M-9_ (3), bla_CTX-M-15_ (1)
**ST273**	147	2	HC (1), HH (1)	17 (2)	bla_SHV_ (1), bla_TEM_ (2), bla_CTX-M_ (2), bla_CTX-M-1_ (2)
**ST412**	412	4	HH (1), W (1), HC (2)	17 (2), 16 (2)	bla_SHV_ (4), bla_TEM_ (4), bla_CTX-M_ (3), bla_CTX-M-1_ (2), bla_CTX-M-9_ (1)
**ST580**	147	2	W (1), HC (1)	16 (1), 17 (1)	bla_TEM_ (2)
**ST859**	11	9	CC (7), RP (1), HH (1)	17 (9)	bla_SHV_ (8), bla_TEM_ (9), bla_CTX-M_ (8), bla_CTX-M-1_ (5), bla_CTX-M-8_ (2), bla_CTX-M-9,_ (4), bla_CTX-M-15_ (3)
**ST1137**	147	6	CC (3), HC (3)	17 (5), 16 (1)	bla_SHV,_ (6), bla_TEM_ (5),bla_CTX-M_ (5), bla_CTX-M-1_ (5), bla_CTX-M-8_ (1), bla_CTX-M-15_ (1)
**ST1561**	147	1	CC (1)	16 (1)	bla_SHV_ (1), bla_TEM_ (1), bla_CTX-M_ (1), bla_CTX-M-9,_ (1)
**ST1709**	147	1	HC (1)	17 (1)	bla_SHV_ (1), bla_CTX-M_ (1), bla_CTX-M-1_ (1), bla_CTX-M-8_ (1)
**ST2167**	37	2	W (2)	17 (2)	bla_SHV_ (2), bla_TEM_ (1), bla_CTX-M_ (2), bla_CTX-M-1_ (2), bla_CTX-M-8_ (2)

**^#^** CB, cow and buffalo; CC, commercial chicken; HC, human clinic; HH, household; RP, rural poultry; SG, sheep and goat; SH, slaughter house; W, water.

## Discussion

The persistently growing AMR crisis is a momentous health threat impacting healthcare systems across the globe. Unwise antimicrobial use (AMU) in human and veterinary medicine is a considerable factor associated with the dissemination of AMR. Likewise, inadequate sewage facilities in densely inhabited areas also play a vital role in AMR dissemination. Strict surveillance is needed for the source track that contributes to the distribution of AMR. To do the needful, the proposed study was conducted to estimate the occurrence of ESBL harboring *K. pneumoniae* in various sample sources.

The current study showed an overall 10% occurrence rate of *K. pneumoniae* among various ecological niches studies in this study. Overall, a non-significant difference was observed among occurrence rates estimated in different sources, i.e., animal (11%), environment (7%), and human (8%) (**Table 1**). Occurrence in animal samples was higher compared to the others. This may be due to the irrational use of antibiotics in livestock, which is a very common practice in low- and middle-income countries like Pakistan, where antibiotics are frequently used as growth promoters in livestock. Our study results are comparable with the findings published from the neighboring country India where they observed a 6% occurrence rate of *K. pneumoniae* among livestock samples (Kar et al., 2015). Likewise, in the past, another study conducted in England reported a somewhat similar occurrence rate of *K. pneumoniae* recovered from veterinary samples (Randall et al. (2014))

Overall, the occurrence of ESBL harboring *K. pneumoniae* was higher in chicken (15.7%) compared to other food animals [sheep and goat (5.0%) and cow and buffalo (2.8%)]. The high occurrence of antibiotic-resistant *K. pneumoniae* among commercial chicken was in line with (25.8) in Norway (Franklin-Alming et al., 2021) and lower in Egypt (Hamza et al., 2016) and Algeria (Chaalal et al., 2021). Antibiotic resistance in sheep and goat (5.0%) and cow and buffalo (2.8%) is lowered compared to commercial chicken in this study. The high prevalence of *K. pneumoniae* in chickens shows that commercial chickens might be the main reservoir of *K. pneumoniae* more than other food animals (Aslam et al., 2018). Therefore, it is vital to explore it in detail to overcome this menace. High AMU in commercial chickens may be another important factor for the presence of high antibiotic resistance *K. pneumoniae* because AMU can influence the broiler gut microbiota to help *K. pneumoniae* for antibiotic resistance development (Torok et al., 2011; Franklin-Alming et al., 2021). Our results are comparable with previous studies’ observations, which also showed a different level of ESBL phenotypes in food animals, including poultry and humans (Ilyas et al., 2021; Zainab et al., 2022). In this study, the prevalence of antibiotic resistance is less compared to another study reported from India and Pakistan (Liebana et al., 2013; Szmolka and Nagy, 2013; Brower et al., 2017; Ilyas et al., 2021). We collected samples from open commercial poultry farms and associated environments in a rural area of Pakistan, which might be the possible reason behind the low antibiotic resistance in this study.

A wide range of antibiotics were used for antimicrobial susceptibility testing. The *K. pneumoniae* isolates showed resistance against all antibiotics except tigecycline, polymyxin, and ceftolozane/tazobactam. Different studies have already reported maximum antibiotic resistance against different antibiotic classes as a study reported from Algeria showed 100% resistance against clavulanic acid/amoxicillin, piperacillin, amoxicillin, tazobactam/piperacillin, ticarcillin, clavulanic acid/ticarcillin, cefixime, cefotaxime, cefoxitime, ceftazidime, and ertapenem, which is in line with our results (Chaalal et al., 2021). Our results are in line with previously reported studies where a similar resistance pattern was observed in *K. pneumoniae* recovered from various sample sources (Grave et al., 2010; Wang et al., 2012; Brower et al., 2017; Ilyas et al., 2021; Zainab et al., 2022). These antibiotics normally do not prescribe for veterinary and animal feed use. Further, these drugs are also not available in rural medicine stores. These factors might be the possible cause of low resistance.

Our results showed that the presence of ESBL genes such as *bla*TEM, *bla*SHV*, bla*CTX-M, *bla*CTX-M-1and *bla*CTX-M-8*, bla*CTX-M-9, and *bla*CTX-M-15 was high in chickens and clinical isolates compared to other isolates. A study reported the dissemination of ESBLs harboring Enterobacterales in veterinary fecal, farm premises, and waste milk samples in England and Wales in 2011 (Randall et al., 2014). Another study has reported *bla*CTX-M in waste milk and highlighted the effect of the farm environment on the transmission of antibiotic resistance gene (ARGs). A similar study in Germany found plasmid-mediated ESBLs producing Gram-negative bacteria in cattle (Freitag et al., 2017). A significant prevalence of *K. pneumoniae* in poultry meat has been reported in China (Wu et al., 2016). Although, they have identified *bla*CTX-M as one of the most prevalent resistance genes in the samples. Some studies reported that *bla*CTX-M and *bla*SHV genes were the most predominant genotype among ESBL genes in chickens (Zarfel et al., 2014). The ESBL resistance genes were commonly reported in single or multiple gene combinations (Randall et al., 2014; Zarfel et al., 2014). In this study, different ESBL resistance genes were seen in multiple combinations in all *K. pneumoniae* isolates, particularly in animal and clinical isolates compared to other isolates, making them MDR bacteria. The presence of MDR-ESBL bacteria is a threat to livestock, humans, and their associated environment, which may limit treatment options for clinicians. Furthermore, MDR-ESBL bacteria prompted the use of those antibiotics, which has stopped due to toxicity issues (Fard, 2004). The high prevalence of ESBLs’ resistance to *K. pneumoniae* suggests the initiation of a national surveillance system to understand its prevalence in food animals and their environment. The presence of ESBL genes in commercial and rural poultry has been reported in previous studies, which are in line with our results (Sarba et al., 2019). In clinical isolates, the presence of *bla*TEM was high compared to *bla*CTX-M-1, *bla*CTX-M-8, *bla*CTX-M-9, and *bla*CTX-M-15, which is different compared to previous studies (Osuna et al., 2016; Rahman et al., 2016; Bilal et al., 2021).

Overall, a total of 18 distinct STs were identified with their specific allelic pattern among various ecological niches. ST29 and ST258 were the most abundant STs followed by ST11, ST859, and ST1137. A total of 28 isolates were classified as ST29 (n = 14) and ST258 (n = 14). Both of these STs were only found in livestock and their environment, which is quite interesting. Overall, due to the unavailability of data about the *K. pneumoniae* ST prevalence in livestock and their associated environments, we cannot compare our results with such studies. Our group has reported *K. pneumoniae* ST 29, ST 258, and ST11 among veterinary and waste samples in Pakistan (Aslam et al., 2020; Chaudhry et al., 2020). In the recent past, sporadic reports of *K. pneumoniae* ST11 have been published in Pakistan (Qamar et al., 2018). The current study revealed a comprehensive status of *K. pneumoniae* ST 29, ST 258, and ST11 due to the molecular resistance pattern depicting various ARGs. Different studies have reported these STs’ prevalence among various sources in the world (Leverstein-van Hall et al., 2011; Uz Zaman et al., 2014; Eibach et al., 2018). In this study, the majority of STs were found in human samples such as ST48, 111, 273, and 412. These types of ST are already reported in human samples, but here, we report some of these STs also in animal samples ST859 and 1137, which is quite interesting.

In conclusion, **t**he occurrence of ESBL harboring *K. pneumoniae* in different “One Health” segments is a serious health threat. Further, sanitation facilities are poor in local settings, which is an important risk factor linked with the dissemination of resistant *K. pneumoniae* in the community. Therefore, effective surveillance and monitoring measures are needed to control this health menace.

## Data availability statement

The original contributions presented in the study are included in the article/**Supplementary Material**. Further inquiries can be directed to the corresponding author.

## Ethics statement

The studies involving human participants were reviewed and approved by Ethics Committee of south china agricultural University. The patients/participants provided their written informed consent to participate in this study. The animal study was reviewed and approved by Ethics Committee of south china agricultural University.

## Author contributions

All authors contributed to the article and approved the submitted version.
