# Supplementary material for: Occurrence of extended- spectrum β-lactamase harboring K. pneumoniae in various sources: a one health perspective
Source: Front Cell Infect Microbiol. 2023 May 23;13:1103319. doi: 10.3389/fcimb.2023.1103319 (PMC10242356; doi:10.3389/fcimb.2023.1103319)
Supplement: Supplementary file 1 [file DataSheet_1.pdf]

Supplimentary Table: The phenotypic antibiotic resistance results

Name	Resistant	Sensitive
Amikacin hydrate (AK)	75(93.7)	5(6.3%)
Cephalexin (CN)	79(98.7)	1(1.3%)
Tobramycin(TOB)	78(97.5)	2(2.5%)
Ampicillin(AMP),	80(100%)	0(0%)
Amoxicillin/clavulanate (AMC)	80(100%)	0(0%)
Piperacillin (PIP)	80(100%)	0(0%)
piperacillin/tazobactam(TZP)	80(100%)	0(0%)
Cefoperazone + Sulbactam (SCF)	80(100%)	0(0%)
Cefepime (FEP)	80(100%)	0(0%)
Cefotaxime (CTX)	80(100%)	0(0%)
Ceftriaxone (CRO)	80(100%)	0(0%)
Imipenem (IMP)	80(100%)	0(0%)
Meropenem (MEM)	80(100%)	0(0%)
Ciprofloxacin (CIP)	80(100%)	0(0%)
Levofloxacin (LEV)	80(100%)	0(0%)
Trimethoprim-sulphamethoxazole (SXT)	79(98.7%)	1(1.3%)
Minocycline (MIN)	74(92.5)	6(7.5%)
Ceftolozane/tazobactam (CT)	1(1.3%)	79(98.7%)
Polymixin (PB)	1(1.3%)	79(98.7%)
Tigecycline (TGC)	0	80(100%)
